# Metronomic Chemotherapy in Ovarian Cancer: Current Scenario and AI‐Integrated Future Strategies

**DOI:** 10.1002/cnr2.70620

**Published:** 2026-07-10

**Authors:** Ravi Chauhan, Suraja Kumar Das, Lakshay Malhotra, Rajeev Goyal, Sameer Mirza, Khushi Nehra, Maysaloun Merhi, Ammira S. Al‐Shabeeb Akil, Shahab Uddin, Shweta Tripathi, Ajaz A. Bhat, Mayank Singh

**Affiliations:** ^1^ Department of Medical Oncology (Lab.) Dr. BRAIRCH, All India Institute of Medical Sciences (AIIMS) New Delhi India; ^2^ Department of Biochemistry Sri Venkateswara College, University of Delhi New Delhi India; ^3^ Department of Biochemistry Lady Harding Medical College New Delhi India; ^4^ Department of Chemistry, College of Sciences United Arab Emirates University Al‐Ain UAE; ^5^ Kasturba Medical College, Manipal Academy of Higher Education (MAHE) Manipal India; ^6^ National Centre for Cancer Care and Research, Hamad Medical Corporation Doha Qatar; ^7^ Department of Human Genetics‐Precision Medicine in Diabetes, Obesity and Cancer Research Program Sidra Medicine Doha Qatar; ^8^ Translational Research Institute, Academic Health System, Hamad Medical Corporation Doha Qatar; ^9^ Laboratory Animal Research Center Qatar University Doha Qatar; ^10^ Department of Biology Tuskegee University Tuskegee Alabama USA

**Keywords:** anti‐VEGF therapy, artificial intelligence, immunomodulation, immunotherapeutic agents, metronomic chemotherapy, ovarian cancer, personalized medicine, predictive modeling, targeted therapies, tumor microenvironment

## Abstract

**Background:**

Ovarian cancer (OC) is a globally prevalent malignancy associated with a high mortality rate and marked biological heterogeneity, with most cases arising from epithelial cells and presenting as serous, endometrioid, or clear cell subtypes. Standard management is largely stage dependent and primarily involves cytoreductive surgery followed by platinum‐ and taxane‐based chemotherapy, with modifications based on disease extent and patient factors.

**Recent Findings:**

In recent years, the therapeutic landscape of OC has evolved with the introduction of maintenance strategies and targeted therapies, particularly driven by advances in molecular profiling and the identification of biomarkers such as BRCA mutations and homologous recombination deficiency (HRD). These developments have led to the clinical integration of PARP inhibitors and anti‐angiogenic agents such as bevacizumab, which have improved disease control and survival outcomes as part of standard treatment strategies in OC, whereas immunotherapeutic approaches remain largely investigational and are currently limited to clinical settings. Metronomic chemotherapy (MCT), characterized by the continuous administration of low‐dose chemotherapeutic agents, has emerged as a promising alternative to conventional maximum tolerated dose regimens. MCT offers reduced systemic toxicity while exerting sustained antitumor effects through modulation of the tumor microenvironment, inhibition of angiogenesis, and enhancement of antitumor immune responses, thereby addressing key limitations of standard chemotherapy, including resistance and cumulative adverse effects. Furthermore, the integration of artificial intelligence (AI) into metronomic treatment strategies holds significant potential for optimizing drug selection, dosing schedules, and patient stratification. AI‐driven tools can facilitate predictive modeling, high‐throughput data analysis, and personalized treatment planning, ultimately enhancing therapeutic efficacy while minimizing toxicity.

**Conclusion:**

This review summarizes current management strategies in OC with particular emphasis on maintenance therapies, targeted approaches, and emerging evidence supporting MCT and AI‐enabled approaches as potential future directions in OC therapy.

**Abbreviations**
AE: autoencoders
AI: artificial intelligence
AKT: Ak strain transforming
ANN: artificial neural network
BEV: oral cyclophosphamide and bevacizumab
BRCA: breast cancer gene
CNN: convolution neural network
COX: cyclooxygenase
CR: complete response
CVNN: complex‐valued neural network
CyTo: topotecan and cyclophosphamide
DL: deep learning
DNN: deep neural network
DRL: deep reinforcement learning
EGFR: epidermal growth factor receptor
EOC: epithelial ovarian cancer
EPC: endothelial progenitor cell
GAN: generative adversarial network
GIP: gastrointestinal perforation
GPB: gemcitabine, platinum, and bevacizumab
HTVS: high‐throughput virtual screening
IP: intraperitonial
JNK: Janus kinase
LBVS: ligand‐based virtual screening
LDM: low‐dose metronomic
LMICs: low‐ and middle‐income countries
LR: linear regression
MCT: metronomic chemotherapy
ML: machine learning
MMPs: matrix metalloproteinases
MOC: metronomic oral cyclophosphamide
MTD: maximum tolerated dose
mTOR: mammalian target of rapamycin
NLP: natural language processing
OC: ovarian cancer
ORR: objective response rate
OS: overall survival
PARP: poly (ADP ribose) polymerase
PDGF: platelet‐derived growth factor
PFS: progression‐free survival
PI3K: phosphatidylinositol 3‐kinase
PPC: primary peritoneal cancer
PR: partial response
PSC: peritoneal serous carcinoma
QSAR: quantitative structure–activity relationship
RF: random forest
RNN: recurrent neural network
RR: response rate
SBVS: structure‐based virtual screening
STAT: signal transducer and activator of transcription
TAA: tumor‐associated antigen
TEC: tumor endothelial cell
VAE: variational autoencoders
VEGF: vascular endothelial growth factor
VS: virtual screening

## 1 Introduction

Ovarian cancer (OC) is the most common gynecological malignancy worldwide and continues to pose a major global health challenge, with the highest mortality rate among women [1]. The disease comprises diverse histological and molecular subtypes, with epithelial OCs, particularly serous, endometrioid, and clear cell carcinomas, accounting for the majority of cases [2, 3]. The management of OC is increasingly personalized, based on disease stage, histologic subtype, molecular profile, and response to prior therapies, but therapeutic stratification remains relatively limited compared to other malignancies such as breast cancer or non‐small lung cancer.

Standard treatment of OC involves cytoreductive surgery followed by platinum‐based chemotherapy with agents such as carboplatin or cisplatin, often combined with taxanes [4, 5, 6]. The addition of targeted therapy like bevacizumab a monoclonal antibody that inhibits vascular endothelial growth factor (VEGF)‐mediated angiogenesis, and poly (ADP‐ribose) polymerase (PARP) inhibitors such as olaparib, rucaparib, and niraparib, which exploit defects in homologous recombination repair by inducing synthetic lethality in tumor cells [7, 8]. In addition, anti‐angiogenic tyrosine kinase inhibitors, such as pazopanib, nintedanib, and sorafenib, have demonstrated clinical activity in OC through inhibition of multiple receptors, including VEGFR, PDGFR, and FGFR, and thereby disrupting tumor vascularization and growth. These agents are not currently considered standard treatment for OC and are used in investigational or selected clinical settings [9, 10].

However, despite these advances, the clinical benefit of current therapies remains limited by the development of treatment resistance, toxicity, and disease recurrence. Conventional maximum tolerated dose (MTD) chemotherapy is effective in achieving initial disease control but is associated with significant systemic toxicity, immunosuppression, and a potential pro‐angiogenic rebound effect, which ultimately contributes to treatment failure over time [11].

Even with the incorporation of targeted agents and combination regimens, a critical gap persists between treatment intensity and long‐term disease control, especially in pretreated patient populations. Consequently, a significant unmet need therefore persists, particularly in recurrent and platinum‐resistant OC, which will require more effective and better‐tolerated doses [12].

In this context, metronomic chemotherapy (MCT) has emerged as an alternative approach, first conceptualized by Kerbel and Hanahan, which involves the frequent, continuous administration of low‐dose chemotherapeutic agents without prolonged drug‐free intervals. Unlike conventional cytotoxic approaches, MCT exerts varied antitumor effects by targeting tumor angiogenesis, modulating immune responses, and influencing the tumor microenvironment rather than tumor cell death, as these mechanisms play an important role in OC progression and resistance [13, 14].

By sustaining continuous pharmacologic exposure at low doses, MCT provides reduced toxicity, improved tolerability, feasibility, and the convenience of oral administration, making it particularly advantageous for patients with recurrent or platinum‐resistant OC as well as for healthcare systems in low‐ and middle‐income countries (LMICs) [15]. By continuously suppressing angiogenesis, modulating immune responses, promoting tumor dormancy, and reshaping stromal interactions, MCT establishes a dynamic antitumor environment that enhances long‐term disease control while minimizing collateral damage to normal tissues [16]. These mechanisms have demonstrated particular relevance in OC, where MCT improves treatment tolerability and enables rational combinations with anti‐angiogenic agents, immune checkpoint inhibitors, and PARP inhibitors, especially in heavily pretreated and platinum‐resistant settings [17].

However, the clinical use of MCT is limited by challenges in dose optimization and patient selection, which defines the need for data‐driven approaches such as artificial intelligence (AI) to enable more precise and effective treatment strategies. Advanced AI methodologies, including machine learning (ML), deep learning (DL), and neural network‐based models, enable the analysis of complex, multidimensional datasets, including genomics, tumor microenvironment characteristics, pharmacokinetics, toxicity profiles, and clinical outcomes [18].

In case of MCT, AI facilitates dose optimization, patient stratification, and prediction of therapeutic response while minimizing toxicity. AI‐driven phenotypic response modeling and high‐throughput in silico screening enable rapid identification of synergistic metronomic combinations, support drug repurposing, and reduce dependence on costly and time‐intensive clinical trials [19]. Moreover, AI enables real‐time adaptive metronomic therapy by integrating longitudinal biomarkers, imaging data, and clinical monitoring to dynamically adjust dosing schedules in response to tumor evolution and host tolerance. AI‐based platforms also improve safety by predicting adverse drug reactions, assessing on‐target and off‐target effects, and guiding long‐term low‐dose treatment decisions [20].

Collectively, the convergence of AI and MCT represents a transformative strategy that addresses biological heterogeneity, improves treatment tolerability, reduces economic burden, and advances precision therapy in OC management.

## 2 MCT and Tumor Microenvironment Remodeling

**FIGURE 1:**
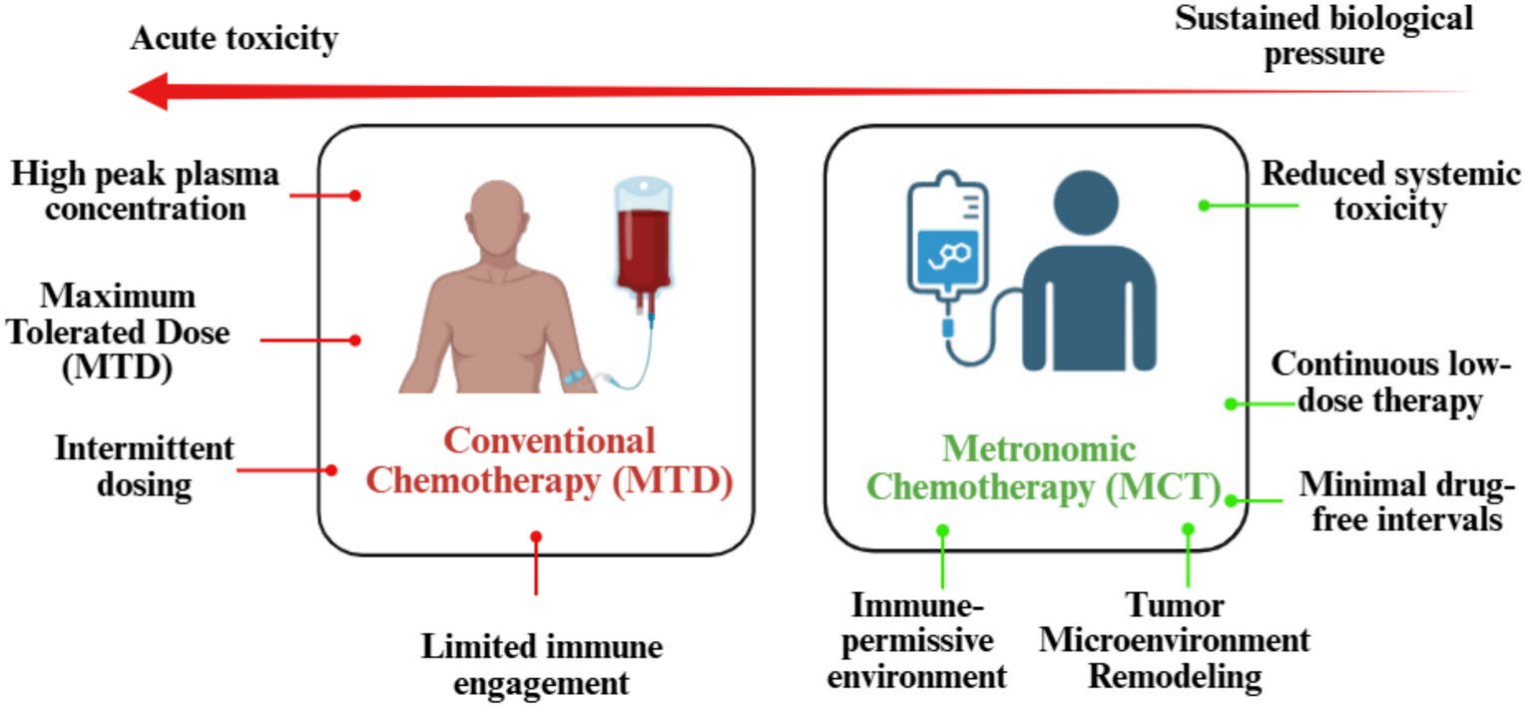
Comparison of conventional maximum tolerated dose (MTD) chemotherapy and metronomic chemotherapy (MCT). The figure contrasts conventional MTD chemotherapy, characterized by intermittent high‐dose administration that produces high peak plasma drug concentrations, acute systemic toxicity, and limited immune engagement, with MCT, which involves continuous low‐dose treatment with minimal drug‐free intervals. MCT maintains sustained biological pressure on the tumor, reduces systemic toxicity, and promotes tumor microenvironment remodeling with improved immune permissiveness, thereby supporting prolonged disease control compared with conventional chemotherapy.

MCT represents a paradigm shift from conventional MTD chemotherapy by focusing on sustained biological pressure rather than transient cytotoxic peaks. As illustrated in Figure 1, conventional MTD chemotherapy relies on intermittent administration of high drug doses, resulting in high peak plasma concentrations and acute systemic toxicity. These regimens require extended drug‐free intervals for patient recovery, which can limit continuous tumor control [11].

In contrast, MCT involves the continuous or frequent administration of low‐dose chemotherapeutic agents with minimal treatment‐free intervals, leading to reduced systemic toxicity and improved tolerability. Importantly, this dosing strategy induces tumor microenvironment remodeling through sustained anti‐angiogenic effects [12].

**FIGURE 2:**
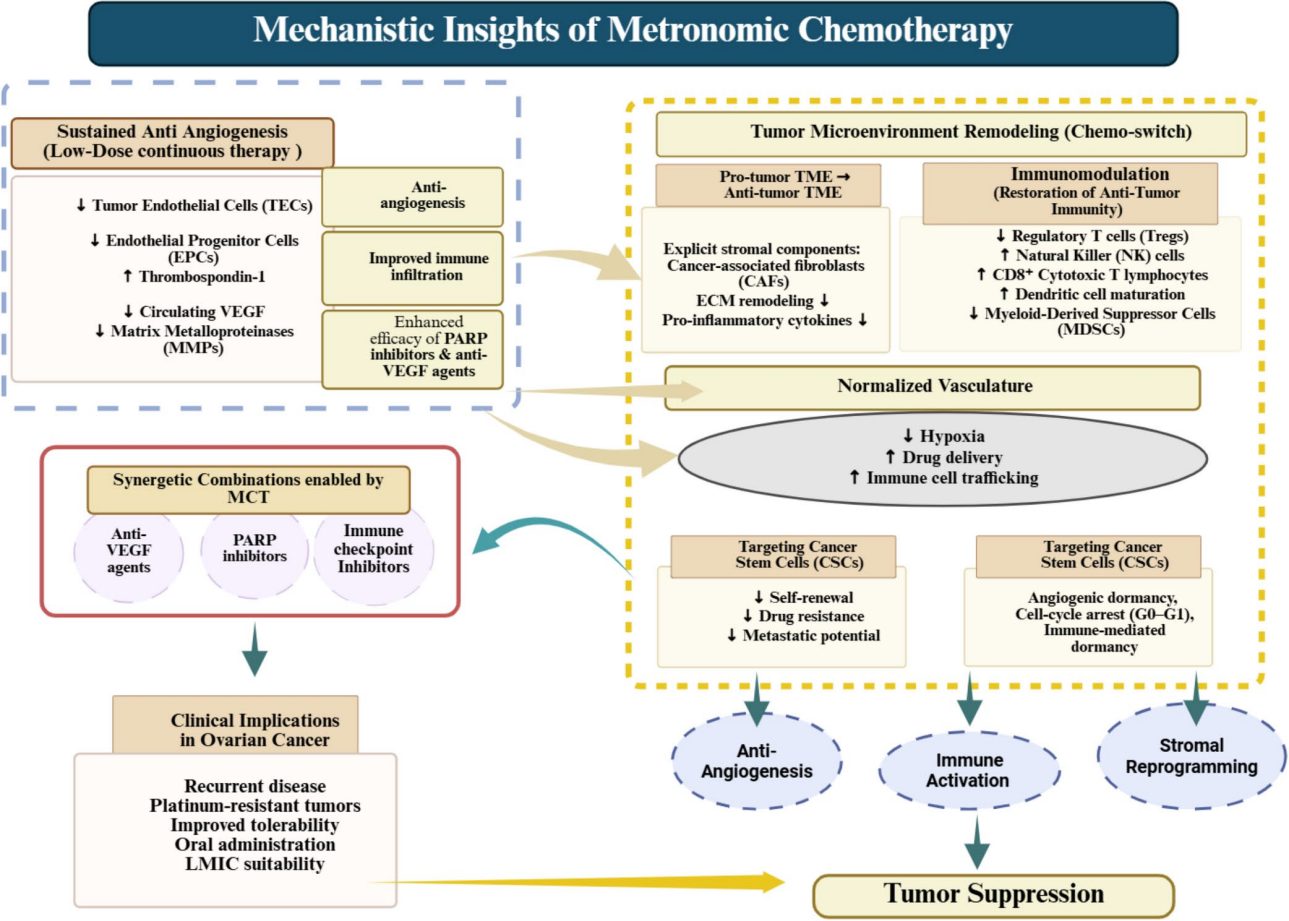
Mechanistic insights of metronomic chemotherapy (MCT). The schematic illustrates the interconnected biological mechanisms underlying MCT. Continuous low‐dose treatment exerts sustained anti‐angiogenic effects by targeting tumor endothelial and endothelial progenitor cells, reducing VEGF and matrix metalloproteinases, and increasing endogenous anti‐angiogenic factors such as thrombospondin‐1. These effects contribute to vascular normalization, reduced hypoxia, improved drug delivery, and enhanced immune cell trafficking. In parallel, MCT induces tumor microenvironment remodeling (chemo‐switch), shifting the milieu from pro‐tumor to antitumor through stromal reprogramming, immunomodulation, and restoration of antitumor immunity. MCT also facilitates synergistic combinations with anti‐VEGF agents, PARP inhibitors, and immune checkpoint inhibitors, and targets cancer stem cell populations to reduce self‐renewal, resistance, and metastatic potential. Collectively, these processes converge on immune activation, stromal reprogramming, and sustained tumor suppression, with important clinical implications for recurrent and platinum‐resistant ovarian cancer.

The principal mechanism of action of MCT is its potent anti‐angiogenic effect, achieved through the continuous administration of low‐dose chemotherapeutic agents that selectively disrupt tumor‐driven blood vessel formation, as illustrated in Figure 2. Unlike monoclonal antibody‐based anti‐VEGF therapies, which directly neutralize VEGF, MCT interferes more broadly with the angiogenic process itself, thereby modulating the tumor vasculature in a sustained and dynamic manner [15].

At the cellular level, MCT primarily targets tumor endothelial cells (TECs) and endothelial progenitor cells (EPCs), leading to suppression of neovascularization essential for tumor growth and survival. This is mediated through the induction of endogenous anti‐angiogenic factors such as thrombospondin‐1, inhibition of pro‐angiogenic signaling molecules, and reduction in circulating VEGF levels [11]. By compromising the tumor blood supply, MCT indirectly induces hypoxia and nutrient deprivation within the tumor microenvironment, resulting in the elimination of both drug‐sensitive and drug‐resistant cancer cell populations [17].

Furthermore, MCT inhibits angiogenesis through multiple complementary pathways, including direct cytotoxic effects on endothelial cells, alteration of the balance between pro‐ and anti‐angiogenic growth factors, depletion of circulating EPCs, and suppression of matrix metalloproteinases (MMPs) that are critical for extracellular matrix remodeling and new vessel formation [21].

Additionally, MCT has been shown to regulate tumor‐associated stromal cells via the “chemo‐switch” concept as illustrated in Figure 2, shifting the tumor microenvironment from a pro‐tumorigenic to an antitumorigenic state. Collectively, these effects demonstrate that MCT can be a sustained therapeutic approach through coordinated modulation of vascular, metabolic, and stromal components of the tumor microenvironment.

## 3 Systemic Immunomodulatory Effects of MCT

Beyond its effects on the tumor microenvironment, MCT exerts systemic immunomodulatory effects that contribute to its overall antitumor efficacy. In contrast to conventional chemotherapy, which is associated with immunosuppression, metronomic dosing has been shown to enhance antitumor immune responses through multiple complementary mechanisms [17].

A key immunomodulatory effect of MCT is the selective depletion of immunosuppressive regulatory T cells (Tregs), which play a critical role in inhibiting effective antitumor immunity. Low‐dose cyclophosphamide has been demonstrated to reduce Treg populations, thereby restoring tumor‐specific immune responses in both preclinical and clinical settings [22]. This reduction promotes the activation of effector immune cells, including natural killer (NK) cells and cytotoxic T lymphocytes, resulting in enhanced immune‐mediated tumor control [17, 23].

In addition, MCT facilitates the maturation and functional activation of dendritic cells, which are essential for antigen presentation and T‐cell activation. Several agents commonly used in metronomic regimens, such as vinblastine, paclitaxel, and etoposide, have been shown to promote dendritic cell differentiation and function, thereby supporting adaptive immune responses as illustrated in Figure 2 [15, 23]. Furthermore, modulation of myeloid‐derived suppressor cells contributes to the reversal of tumor‐induced immune suppression, reinforcing the immunostimulatory effects of this therapeutic approach [24].

Despite these encouraging findings, the precise mechanisms underlying immune modulation by MCT remain incompletely understood. Interpretation of immune‐related effects is further complicated by studies conducted in immunodeficient models, where the absence of functional immune components may underestimate their full immunomodulatory potential [25]. Although durable responses have been reported, tumor relapse may still occur due to the emergence of resistance mechanisms or inadequate immune surveillance [26]. Collectively, these observations suggest that the contribution of the immune system to the efficacy of MCT is variable and context‐dependent.

## 4 Tumor Dormancy in MCT

Tumor dormancy represents a dynamic state regulated by interactions between tumor cells, the tumor microenvironment, and immune surveillance, which is influenced by MCT through its ability to limit cancer cell proliferation and sustain long‐term growth control [27, 28]. MCT can induce cellular senescence and support immune‐mediated tumor suppression and tumor immunogenicity [17]. By continuously suppressing angiogenesis and reducing immune tolerance, MCT creates conditions that favor tumor dormancy, a state that is tightly regulated by immune surveillance and the balance between tumor cell proliferation and cell death [11].

Tumor dormancy arises from dynamic interactions between cancer cells and their microenvironment and can manifest as angiogenic dormancy, cellular dormancy characterized by G0–G1 cell‐cycle arrest, or immune‐mediated dormancy maintained by ongoing immune control [29]. While the precise mechanisms through which MCT induces tumor dormancy are not yet fully understood, emerging evidence suggests that its ability to modulate angiogenesis, immune responses, stromal interactions, and cancer stem cell signaling may collectively contribute to the induction and maintenance of a dormant state. Recent studies further propose that sustaining tumor dormancy, rather than complete tumor eradication, may represent a viable long‐term therapeutic strategy, particularly for preventing relapse and delaying disease progression in treatment‐resistant cancers [30].

## 5 Safety Profile of MCT

Across various cancer types, MCT has been extensively investigated in clinical trials and is generally associated with a more favorable toxicity profile compared with conventional MTD chemotherapy. The continuous administration of low‐dose chemotherapeutic agents enables sustained antitumor effects while minimizing the occurrence of severe acute toxicities. However, the type and severity of adverse effects differ across studies, indicating that the safety profile of MCT is influenced by the specific drug, dosing schedule, combination strategy, and patient characteristics. These differences underscore the importance of systematic toxicity evaluation and individualized treatment planning [17, 31].

Metronomic oral cyclophosphamide (MOC), one of the most frequently used agents in metronomic regimens, has consistently shown lower rates of severe adverse events compared with standard chemotherapy protocols. Nonetheless, low‐grade toxicities, including mucositis and thrombocytopenia, have been reported, particularly in palliative care settings, suggesting that long‐term administration may still present clinical challenges [32]. Similar variability in toxicity has been noted with other metronomic agents and combination regimens, emphasizing the need for careful patient selection and ongoing monitoring.

In OC, MCT has shown the potential to achieve effective disease control with reduced toxicity compared with conventional approaches. Oral cyclophosphamide combined with bevacizumab in recurrent OC is mainly associated with Grade 2 toxicities, with adverse events such as elevated liver enzymes, leukopenia, diarrhea, and fatigue, and occasional serious events reported [33, 34]. Variability in patient responses, including cases of severe anemia and non‐hematological toxicity, has been observed. While bevacizumab provides clinical benefit, it is linked to hypertension and rare gastrointestinal complications, requiring close monitoring [35].

Similar mild to moderate toxicities are seen with weekly topotecan plus biweekly bevacizumab, though neutropenia, hypertension, and gastrointestinal adverse events have been reported. Low‐dose oral topotecan has been associated with dosing‐related challenges, commonly causing anemia and fatigue, along with both hematological and non‐hematological toxicities [21]. Despite progress in combination regimens involving gemcitabine, platinum agents, and bevacizumab, serious adverse events such as bowel perforations and hematologic complications remain concerns [36]. In contrast, bevacizumab combined with oral cyclophosphamide has demonstrated clinical benefit with manageable toxicity [37, 38].

Thalidomide‐based palliative chemotherapy has shown outcomes comparable to standard intravenous therapy, though with a modest reduction in CA‐125 levels [39]. Overall, the wide range of toxicity profiles observed across MCT studies highlights the importance of individualized treatment planning. Careful dose optimization, appropriate patient selection, and vigilant monitoring are essential to maximize therapeutic benefit while minimizing adverse effects, supporting the safe and effective use of MCT in OC.

## 6 Preclinical Models and Therapeutic Strategies in MCT for OC

Preclinical research in OC encompasses both in vitro and in vivo approaches, which together provide complementary insights into tumor biology and therapeutic response. In vitro studies have been instrumental in elucidating the molecular mechanisms underlying OC progression, including dysregulation of key signaling pathways such as PI3K/AKT/mTOR, JAK/STAT, and Wnt/β‐catenin [3, 40]. These investigations have facilitated the development of targeted therapeutic strategies, including pathway‐specific inhibitors and apoptosis‐inducing agents, which suppress tumor growth, invasion, and metastasis [41].

Agents such as enzastaurin and metformin have demonstrated antitumor activity in preclinical models by inducing regulated cell death [40, 42]. In addition, anti‐angiogenic strategies have evolved from in vitro validation to clinical application, with VEGF‐targeting agents such as bevacizumab playing a key role in inhibiting tumor angiogenesis [43]. Similarly, targeting receptor tyrosine kinases within the ErbB family, including EGFR and HER2, using agents such as trastuzumab, has expanded therapeutic options [44]. Other approaches, including proteasome inhibition, microtubule‐targeting agents, and therapies directed against tumor‐associated markers such as CA125, further illustrate the broad impact of in vitro studies in shaping OC therapeutics [3, 6].

Despite these advances, in vitro systems are inherently limited by their inability to replicate the complexity of tumor–host interactions, the tumor microenvironment, and systemic therapeutic responses. Therefore, disease‐relevant animal models are essential for validating these findings in a physiologically relevant context and for assessing the translational potential of therapeutic strategies [16].

Several in vivo studies have explored metronomic and combination‐based approaches in OC. Hashimoto et al. demonstrated that low‐dose metronomic oral topotecan produced significant antitumor activity in a murine model of ovarian carcinoma. Furthermore, when combined with the anti‐angiogenic agent pazopanib, this regimen resulted in a 100% survival rate in treated mice, indicating strong potential for clinical translation [45].

In another study, vandetanib, a dual inhibitor of VEGF and EGFR signaling, enhanced the antitumor efficacy of paclitaxel in ovarian carcinoma xenograft models. Although vandetanib did not significantly alter tumor vessel density, it improved the therapeutic response to paclitaxel, supporting its consideration for future clinical trials [46].

In another study, ABT‐510, a thrombospondin‐1 mimetic peptide, demonstrated a significant ability to reduce tumor burden, ascites formation, and secondary lesion development in epithelial ovarian cancer (EOC). Treatment with ABT‐510 led to vascular remodeling, increased apoptosis, and reduced expression of cell survival proteins, highlighting its therapeutic potential in EOC [47].

Moreover, the combination of ABT‐510 with conventional chemotherapy agents, cisplatin and paclitaxel, improved drug uptake and promoted tumor regression in a mouse model of OC, suggesting its role in enhancing standard chemotherapy regimens [48]. Moreover, Reyes et al. reported that celecoxib, a COX inhibitor, suppressed cell growth and proliferation and induced apoptosis in OC cells in a dose‐dependent manner [49]. However, Bijman et al. observed antagonistic interactions between celecoxib and cisplatin, irrespective of COX‐2 expression levels, indicating that caution is required when considering this combination for OC therapy [50].

Overall, these preclinical studies highlight promising metronomic and combination‐based strategies for OC treatment but emphasize the need for further investigation to determine their clinical relevance and therapeutic benefit.

## 7 Clinical Study‐Based Approaches to MCT Approaches in OC

MCT has emerged as a promising therapeutic strategy in OC, particularly in the recurrent setting, following the widespread use of conventional treatment approaches such as platinum‐based chemotherapy, taxane combinations, and targeted therapies including bevacizumab. While these standard regimens have demonstrated significant clinical benefit, their long‐term utility is often constrained by cumulative toxicity and the eventual development of therapeutic resistance. In this context, MCT aims to improve treatment effectiveness while reducing toxicity by using continuous low‐dose drug administration and by modulating the tumor microenvironment [15].

Although conventional platinum–taxane combinations and bevacizumab‐based regimens are not strictly metronomic in design, they provide important clinical context for the evolution of alternative therapeutic strategies in OC. Early clinical studies evaluating bevacizumab as a single agent and in combination with chemotherapy in recurrent OC demonstrated meaningful antitumor activity and established VEGF inhibition as a key therapeutic approach. In 2007, Burger et al. conducted a Phase II trial evaluating single‐agent bevacizumab in patients with persistent or recurrent EOC or primary peritoneal cancer (PPC), where 21% of patients experienced clinical responses and 40.3% achieved progression‐free survival (PFS) of at least 6 months, with median PFS and overall survival (OS) of 4.7 and 17 months, respectively, indicating promising activity as a second‐ or third‐line therapy [51].

In a separate Phase II study in the same year, Cannistra et al. evaluated bevacizumab in platinum‐resistant EOC or peritoneal serous carcinoma (PSC), reporting partial responses in 15.9% of patients and a median PFS of 4.4 months, although Grade 3–4 adverse events such as hypertension, proteinuria, and gastrointestinal perforation were observed [52]. Furthermore, the AURELIA trial in 2014 demonstrated that the addition of bevacizumab to chemotherapy significantly improved PFS and objective response rate (ORR) in platinum‐resistant OC, although no significant OS benefit was observed, with increased incidence of hypertension, proteinuria, and rare gastrointestinal perforations [53]. Additionally, Richardson et al. reported a high response rate with a gemcitabine‐platinum‐bevacizumab regimen in recurrent OC, although significant toxicities such as bowel perforation were observed [54]. Similarly, a Phase III trial demonstrated that the addition of bevacizumab to standard platinum‐based chemotherapy improved PFS, albeit with increased hypertension [55]. In parallel, modified dosing strategies such as paclitaxel‐platinum regimens in resource‐limited settings and weekly paclitaxel or carboplatin schedules in patients unfit for standard therapy have shown feasibility and clinical benefit, though these represent dose‐dense rather than true metronomic approaches [56, 57].

Collectively, these approaches share mechanistic overlap with MCT, particularly through sustained inhibition of tumor angiogenesis and improved treatment tolerability, thereby providing a strong rationale for the development of metronomic treatment strategies.

Between 2007 and 2025, several studies evaluated different MCT strategies for the management of recurrent OC. These investigations focused on the role of MCT in overcoming the therapeutic challenges associated with recurrent disease. Metronomic oral single‐agent cyclophosphamide (MOC) emerged as an effective and well‐tolerated treatment option for patients with platinum‐pretreated advanced ovarian carcinoma, particularly for those with limited tolerance to high‐dose chemotherapy [58].

The frequent incorporation of cyclophosphamide in metronomic regimens is attributed to its suitability for continuous low‐dose administration, supported by favorable pharmacokinetics, oral bioavailability, and a manageable toxicity profile. Beyond its cytotoxic effects, it exhibits anti‐angiogenic activity by inhibiting endothelial cell proliferation and reducing pro‐angiogenic factors such as VEGF. Additionally, its immunomodulatory properties, including selective depletion of regulatory T cells, contribute to enhanced antitumor immune responses. These combined effects support its role as a backbone agent in MCT [15].

In addition, combination metronomic regimens incorporating agents such as etoposide, cyclophosphamide, tamoxifen, bevacizumab, pembrolizumab, and topotecan demonstrated encouraging response rates with acceptable toxicity profiles, making them suitable options for patients who are unfit for intensive intravenous chemotherapy [59, 60].

Bevacizumab, a VEGF‐targeting monoclonal antibody, and tyrosine kinase inhibitors such as pazopanib and nintedanib, which inhibit VEGFR and PDGFR, FGFR signaling, have shown meaningful clinical activity in metronomic combinations [61]. In parallel, pembrolizumab, a programmed cell death‐1 (PD) inhibitor, contributes to immune‐mediated antitumor effects [59]. These approaches highlight the integration of cytotoxic, anti‐angiogenic, and immunomodulatory strategies in MCT. The inclusion of cytotoxic agents such as topotecan and etoposide in metronomic strategies is based on their compatibility with low‐dose, continuous administration and their complementary mechanisms of action. Topotecan, a topoisomerase I inhibitor, induces DNA strand breaks during replication, whereas etoposide, a topoisomerase II inhibitor, disrupts DNA repair and cell cycle progression, leading to apoptosis in proliferating tumor cells [15].

At metronomic doses, both agents may also exert anti‐angiogenic effects by inhibiting endothelial cell proliferation and disrupting tumor‐associated vasculature. Their well‐characterized pharmacokinetics and feasibility for low‐dose scheduling further support their use in combination regimens. Based on these mechanistic and clinical insights, the following sections provide a detailed overview of specific MCT approaches in OC, including metronomic cyclophosphamide monotherapy and metronomic combination strategies further categorized into anti‐angiogenic combinations, immunotherapy‐based metronomic regimens, and cytotoxic metronomic combinations [15].

### 7.1 Metronomic Cyclophosphamide Monotherapy

Metronomic cyclophosphamide, an alkylating agent that exerts cytotoxic effects through DNA crosslinking and also demonstrates immunomodulatory and anti‐angiogenic properties at low continuous doses, represents one of the earliest and most extensively studied approaches in OC. In 2007, Samaritani et al. reported the first use of low‐dose oral cyclophosphamide as a palliative treatment in a young patient with advanced, platinum‐resistant OC and poor performance status. Despite prior treatment failure with conventional chemotherapy, the patient experienced an impressive PFS of 65 months on daily low‐dose cyclophosphamide, without any reported adverse effects. This study highlighted the potential of low‐dose oral cyclophosphamide as a therapeutic option for platinum‐resistant patients with limited performance status [62].

Subsequent studies have evaluated MOC as both monotherapy and in combination regimens, as mentioned in Table 1. A multicenter retrospective study assessed MOC as salvage therapy in heavily treated recurrent OC patients. The study reported an ORR of 20.4%, with some patients maintaining stable disease for over 6 months. Median PFS was 4 months, and OS was 13 months, with minimal toxicity observed, suggesting MOC as a viable palliative option [63].

**TABLE 1:** Summary of metronomic chemotherapy studies in ovarian cancer.

Study, year	Sample size, patient setting	Treatment regimen	Category	Clinical outcomes
Samaritani et al., 2007 [62]	*N* = 1, recurrent (platinum‐resistant)	Oral cyclophosphamide	Monotherapy	PFS: 65 mo; no toxicity; stable disease
Ferrandina et al., 2014 [63]	*N* = 54, recurrent	Oral cyclophosphamide	Monotherapy	ORR: 20.4%; PFS: 4 months; OS: 13 months; low toxicity
Gebbia et al., 2023 [58]	*N* = 62, recurrent	Oral cyclophosphamide	Monotherapy	ORR: 23%; DCR: 47%; PFS: 3.5 months
Spiliopoulou et al., 2021 [64]	*N* = 68, recurrent	Oral cyclophosphamide	Monotherapy	PFS: 2.6 months; OS: 6 months; clinical benefit: 48%
Chura et al., 2007 [37]	*N* = 15, recurrent	Cyclophosphamide + bevacizumab	Anti‐angiogenic combination	ORR: 53.3%; SD: 20%; PD: 26.7%
Jurado et al., 2008 [65]	*N* = 9, recurrent	Cyclophosphamide + bevacizumab	Anti‐angiogenic combination	ORR: 44%; PFS: 5.5 months; 6 months PFS: 33%; CR: 22.2%; SD: 22.2%; PD: 33.3%
Sanchez‐Munoz et al., 2010 [66]	*N* = 38, recurrent	Cyclophosphamide + bevacizumab	Anti‐angiogenic combination	ORR: 40.5%; PFS: 4.5 months; OS: 10.7 months; 39% ≥ 6‐month PFS
Goto et al., 2023 [33]	*N* = 20, recurrent	Cyclophosphamide + bevacizumab	Anti‐angiogenic	ORR: 7.1%; PFS: 5.3 months; clinical benefit: 36.3%
Barber et al., 2013 [38]	*N* = 66, recurrent	Cyclophosphamide + bevacizumab	Anti‐angiogenic	ORR: 42.4%; improved PFS/OS; well tolerated
Dinkic et al., 2017 [67]	*N* = 16, recurrent (platinum‐resistant)	Cyclophosphamide + pazopanib	Targeted anti‐angiogenic	PFS: 8.35 months; OS: 24.95 months; manageable toxicity
Sharma et al., 2021 [61]	*N* = 75, recurrent (platinum‐resistant)	Cyclophosphamide + etoposide ± pazopanib	Anti‐angiogenic	PFS: 3.4 vs. 5.1 months; OS: 11.2 months
Gupta et al., 2019 [68]	*N* = 52, recurrent	Cyclophosphamide + celecoxib	Anti‐angiogenic	OS: 9.69 vs. 12.5 months; PR: 4% (both arms); SD ≥ 6 months: 12% vs. 19%
Zsiros et al., 2021 [59]	*N* = 40, recurrent	Cyclophosphamide + bevacizumab + pembrolizumab	Immunotherapy combination	ORR: 47.5%; clinical benefit: 95%; durable response: 25%; PFS: 10 months
Steck et al., 2025 [69]	*N* = 19, platinum‐resistant	Pembrolizumab + bevacizumab + cyclophosphamide	Immunotherapy	ORR: 21.1%; clinical benefit: 42.1%; PFS: 4.0 months; OS: 17.0 months; well tolerated
Wysocki et al., 2023 [60]	*N* = 72, recurrent	Topotecan ± cyclophosphamide (CyTo)	Cytotoxic combination	ORR: 27%; PFS: 3.65 months; responders PFS: 10.7 months; better outcomes in overweight/obese
Harsh et al., 2023 [32]	*N* = 40, recurrent/metastatic	Cyclophosphamide + etoposide + tamoxifen	Cytotoxic combination	ORR: 12.5%; DCR: 60%; PFS: 3.7 months; OS: 6.5 months
Attianese et al., 2024 [70]	*N* = 38, recurrent	Cyclophosphamide ± capecitabine	Cytotoxic combination	ORR: 8.6%; SD: 40%; DoR: 7.4 months; clinical benefit (disease stabilization); no Grade 3–4 toxicity
Küçüköner et al., 2012 [71]	*N* = 51, platinum‐resistant	Oral etoposide	Non‐cyclophosphamide	ORR: 17.6%; DCR: 43.1%; PFS: 3.9 months; OS: 16.4 months; manageable toxicity
Downs et al., 2008 [39]	*N* = 69, platinum‐resistant	Topotecan ± thalidomide	Non‐cyclophosphamide	ORR: 21% vs. 47%; PFS: 4 vs. 6 months; OS: 15 vs. 19 months
Comander and Cannistra, 2008 [72]	*N* = 13, maintenance	Oral topotecan	Non‐cyclophosphamide	Poor tolerance; anemia, fatigue; limited feasibility
Ramasubbaiah et al., 2011 [73]	*N* = 69, platinum‐resistant	Sorafenib + topotecan	Non‐cyclophosphamide	ORR: 21% vs. 47%; PFS: 4 vs. 6 months; OS: 15 vs. 19 months

Similarly, real‐world retrospective analysis conducted in Italy evaluated single‐agent MOC in 62 platinum‐pretreated advanced ovarian carcinoma patients, demonstrating an overall response rate (ORR) of 23% and a disease control rate of 47%. Patients with low‐grade indolent tumors responded particularly well, and the treatment was well‐tolerated, underscoring its potential as a palliative option for patients with poor tolerance to high‐dose regimens or extensive prior therapy [58].

Furthermore, in 2021, another study investigated predictors of response in recurrent OC patients receiving oral metronomic cyclophosphamide. Using combined radiological and CA125 criteria, 32% of patients achieved a partial response, 16% had stable disease, and 52% experienced progressive disease. Patients harboring BRCA1/2 mutations showed improved PFS and OS compared to non‐carriers, indicating potential benefits for specific subgroups as mentioned in Table 1 [64].

### 7.2 Metronomic Cyclophosphamide Combination Strategies

#### 7.2.1 Anti‐Angiogenic Combinations

Combination strategies have been extensively explored to enhance therapeutic efficacy. Early studies between 2007 and 2010 demonstrated encouraging outcomes with the combination of oral cyclophosphamide and bevacizumab, a monoclonal antibody targeting VEGF and thereby inhibiting tumor angiogenesis, as mentioned in Table 1. Chura et al. reported a response rate of 53% in 15 patients, including two complete responses and six partial responses, with a median duration of response of 3.9 months and no severe toxicity [37]. Another small study involving nine platinum‐resistant patients demonstrated a 44% ORR, 33% 6‐month PFS, and favorable safety [65]. Another retrospective study of 38 heavily pretreated recurrent OC patients reported an ORR of 40.5%, median PFS of 4.5 months, and OS of 10.7 months, with manageable Grade 3–4 toxicities and no treatment‐related deaths [66]. Another study combining oral cyclophosphamide with bevacizumab in recurrent ovarian and peritoneal cancer (2014–2020) showed a clinical benefit in 36.3% of patients, with median PFS of 5.3 months and OS of 9.2 months. Toxicities were manageable, making this combination suitable for patients challenging to treat after second‐line chemotherapy [33].

In 2013, Barber et al. assessed the combination of intravenous bevacizumab and oral cyclophosphamide in heavily pretreated patients with recurrent ovarian carcinoma. The regimen achieved an ORR of 42.4%, including complete and partial responses, and improved PFS and OS in responders. The treatment was generally well‐tolerated, although some patients required discontinuation due to adverse events [38].

Other combinations have incorporated anti‐angiogenic and targeted agents, as mentioned in Table 1. The PACOVAR trial in 2017 evaluated pazopanib, a multi‐targeted tyrosine kinase inhibitor acting on VEGFR, PDGFR, and c‐KIT pathways, combined with metronomic cyclophosphamide in 16 patients with platinum‐resistant, recurrent OC. The regimen achieved a median PFS of 8.35 months and OS of 24.95 months, with adverse events including liver enzyme elevation, leukopenia, diarrhea, and fatigue, indicating promising activity in this challenging population [67].

Similarly, a Phase II randomized trial conducted at AIIMS, India, by Kumar et al. evaluated pazopanib‐based oral metronomic therapy in 75 patients with platinum‐resistant or refractory EOC. The study compared two treatment arms: Arm A (etoposide + cyclophosphamide) and Arm B (pazopanib + etoposide + cyclophosphamide). The addition of pazopanib in Arm B resulted in a longer median PFS (5.1 months vs. 3.4 months in Arm A), albeit with a slightly higher incidence of oral mucositis and fatigue, suggesting that pazopanib incorporation may represent a promising metronomic strategy [61].

Additional targeted combinations have been evaluated with variable outcomes. Nintedanib, a triple angiokinase inhibitor targeting VEGFR, FGFR, and PDGFR pathways, and celecoxib, a COX‐2 inhibitor with anti‐inflammatory and anti‐angiogenic properties, were assessed in combination with cyclophosphamide; however, these regimens did not demonstrate significant improvements in survival outcomes, although they were generally well‐tolerated as mentioned in Table 1 [34, 68].

#### 7.2.2 Immunotherapy Combinations

Immunotherapy‐based approaches have also shown promising activity. A Phase II trial evaluating pembrolizumab, a PD‐1 immune checkpoint inhibitor, in combination with bevacizumab and oral metronomic cyclophosphamide demonstrated clinical benefit in 95% of patients, including durable responses in 25%, with manageable toxicities such as hypertension, lymphopenia, fatigue, and diarrhea, as mentioned in Table 1 [59].

More recently, a triplet regimen combining pembrolizumab, bevacizumab, and oral metronomic cyclophosphamide demonstrated clinical benefit in heavily pretreated platinum‐resistant OC patients, with an ORR of 21.1% and overall clinical benefit observed in 42.1% of patients, along with manageable toxicity [69].

#### 7.2.3 Cytotoxic Combinations

Additional cyclophosphamide‐based regimens have included combinations with topotecan (CyTo regimen), a topoisomerase I inhibitor, which demonstrated modest but clinically meaningful activity with improved outcomes in selected patient subgroups [60]. In a cohort of 72 pretreated OC patients, metronomic topotecan, administered alone or in combination with cyclophosphamide, resulted in a median PFS of 3.65 months, while patients achieving a biochemical response experienced a prolonged PFS of 10.7 months. Notably, improved outcomes were observed in overweight or obese patients, supporting further evaluation of the CyTo regimen in Phase II trials [60].

Similarly, triple oral metronomic regimens combining etoposide (a topoisomerase II inhibitor), cyclophosphamide, and tamoxifen (a selective estrogen receptor modulator) have demonstrated clinical benefit with symptomatic improvement and acceptable toxicity profiles [32]. In a study involving 40 patients with recurrent or metastatic EOC, this combination achieved a median OS of 6.5 months and a median PFS of 3.7 months. Treatment‐related toxicities included Grade I/II mucositis in 47.5% of patients, Grade III/IV mucositis in 22.5%, and Grade III/IV thrombocytopenia in 22%, with dose reductions required in 37.5% of patients [32].

Furthermore, low‐dose cyclophosphamide with or without capecitabine, an oral fluoropyrimidine inhibiting DNA synthesis, has demonstrated clinical activity and good tolerability in recurrent OC, including elderly and heavily pretreated patients. Studies have reported median PFS of 7–9 months, with notable rates of disease stabilization and symptomatic improvement, even among platinum‐refractory patients, and minimal high‐grade toxicities [70]. In a small prospective cohort of patients with platinum‐resistant recurrent OC, MOC was well‐tolerated, preserved health‐related quality of life, and showed modest benefits in PFS and OS [74].

Collectively, the accumulated clinical evidence demonstrates that metronomic cyclophosphamide, as monotherapy or in rational combinations with anti‐angiogenic, targeted, or immunotherapeutic agents, provides a well‐tolerated and clinically meaningful treatment option for recurrent and platinum‐resistant OC, particularly in patients with poor performance status or those unsuitable for intensive chemotherapy, as described in Table 1.

### 7.3 Other MCT Regimens

Non‐cyclophosphamide metronomic regimens, particularly those based on etoposide and topotecan, have also been explored in OC as described in Table 1. Küçüköner et al. investigated long‐term, low‐dose oral etoposide, a topoisomerase II inhibitor that induces DNA strand breaks as a treatment option for platinum‐resistant EOC. Among 51 platinum‐resistant patients, 17.6% achieved a partial response, while 25.5% had stable disease. The median PFS was 3.9 months, and the median OS was 16.4 months. Hematologic and gastrointestinal toxicities were observed in 37.3% and 29.4% of patients, respectively. The study concluded that chronic low‐dose oral etoposide is generally effective and well‐tolerated in platinum‐resistant OC patients [71].

Topotecan‐based approaches have also been evaluated in a prospective randomized Phase II trial. Downs Jr. et al. compared topotecan alone to topotecan combined with thalidomide in 69 women with platinum‐resistant recurrent EOC. The thalidomide arm demonstrated higher ORRs (47% vs. 21%), longer median PFS (6 months vs. 4 months), and improved median OS (19 months vs. 15 months) compared to the control arm, with similar toxicity profiles. The study concluded that adding thalidomide to topotecan improves response rates and warrants further investigation in larger studies [39].

Moreover, Comander and Cannistra evaluated oral maintenance topotecan in patients with advanced ovarian, fallopian tube, or primary peritoneal serous cancer who had achieved a complete clinical response after platinum‐based chemotherapy. Oral topotecan administered in escalating doses was poorly tolerated due to progressive anemia and fatigue, with most patients requiring dose reductions and many unable to complete six planned cycles. Hematologic toxicity and diarrhea were the most common adverse events, indicating that the tested dose and schedule of oral topotecan is not feasible in this patient population [72].

Additionally, Ramasubbaiah et al. assessed the combination of sorafenib with weekly topotecan in patients with platinum‐resistant OC or primary peritoneal carcinomatosis. While the regimen demonstrated modest efficacy, with 16.7% partial responses and 46.7% stable disease, significant toxicities, including hematologic adverse events and fatigue, led to frequent dose reductions, limiting its clinical applicability [73].

Although primarily cyclophosphamide‐based, some metronomic regimens incorporating etoposide have also been explored. In patients with recurrent or metastatic OC unfit for intensive intravenous chemotherapy, a triple oral metronomic regimen of etoposide, cyclophosphamide, and tamoxifen demonstrated clinical activity, with 60% of patients achieving disease control, including 12.5% partial responses and 47.5% stable disease. Median PFS and OS were 3.7 and 6.5 months, respectively. Treatment‐related toxicities included Grade III/IV mucositis and thrombocytopenia, with dose reductions required in 37.5% of patients [32].

In summary, metronomic regimens based on etoposide and topotecan offer modest disease control with manageable toxicity in selected patients with platinum‐resistant OC. However, their overall clinical benefit remains limited, underscoring the need for optimized dosing strategies and further validation in larger prospective trials mentioned in Table 1.

## 8 Potential Application of AI in MCT

AI, including ML and DL approaches, has rapidly transformed modern medicine by enabling data‐driven decision making, improving diagnostic accuracy, predicting disease outcomes, and drug development [18, 20]. AI has also expanded into cancer research by simplifying complex biological processes, optimizing resource utilization, reducing clinical risks, and minimizing costs, manpower, time, and ethical concerns [75, 76].

In OC, AI‐driven ML and radiomics models have demonstrated increasing clinical relevance in predicting treatment response, disease progression, and survival outcomes through the integration of imaging and clinical features. For example, Hatamikia et al. have developed predictive and robust radiomics models for chemotherapy response in high‐grade serous ovarian carcinoma, highlighting the potential of AI to integrate imaging‐derived features with clinical parameters for improved treatment stratification [77] Additional studies have also explored AI‐assisted histopathological analysis, tumor segmentation, and biomarker integrated predictive modeling in OC, further supporting the emerging role of AI in precision oncology [78].

However, despite these advances, the direct application of AI specifically for MCT in OC remains limited, and most proposed strategies are currently conceptual. Existing evidence provides a promising framework for the future integration of AI into MCT by supporting dose optimization, patient selection, and toxicity prediction through analysis of multidimensional clinical and molecular data.

MCT, a regimen of continuous low‐dose chemotherapy targeting tumor angiogenesis and modulating the immune microenvironment with reduced toxicity compared with conventional MTD schedules, presents significant opportunities for integration with AI due to the complexity of optimizing dosing schedules, patient selection, and combination strategies across heterogeneous tumors such as OC. AI can analyze multidimensional datasets, including tumor genetic profiles, prior treatment responses, clinical parameters, and real‐time patient monitoring, to develop predictive models for personalized metronomic regimens, minimizing toxicity while improving efficacy [79, 80].

Moreover, AI‐driven phenotypic response surface modeling allows optimization of multidrug combinations and dosing strategies by fitting response surfaces to drug interactions and biomarker values, enabling rapid, patient‐specific therapy adaptation without extensive empirical screening [81]. These approaches can refine metronomic combinations; for example, cyclophosphamide with targeted agents, bevacizumab, or immunotherapies by predicting synergistic interactions and identifying optimal therapeutic windows. High‐throughput in silico screening powered by AI further accelerates the discovery of novel metronomic regimens, supports drug repurposing, predicts drug‐target interactions, and reduces reliance on costly and time‐consuming clinical trials [82, 83, 84].

AI also enables real‐time adaptive therapy by integrating longitudinal data, including biomarkers, imaging, and wearable device outputs, allowing dynamic adjustment of metronomic doses in response to tumor progression and host response [81]. Such adaptive approaches may enhance sustained disease control while reducing toxicity, which is especially critical in OC patients who are often heavily pretreated, platinum‐resistant, or have compromised performance status. AI tools can also predict adverse drug reactions and toxicity risks with high sensitivity, assisting clinicians in balancing efficacy and safety in long‐term, low‐dose regimens [85, 86]. In addition, AI‐assisted multi‐omics analysis can support biomarker discovery, identify resistance mechanisms, and improve patient selection for personalized metronomic combination treatment strategies in OC.

**FIGURE 3:**
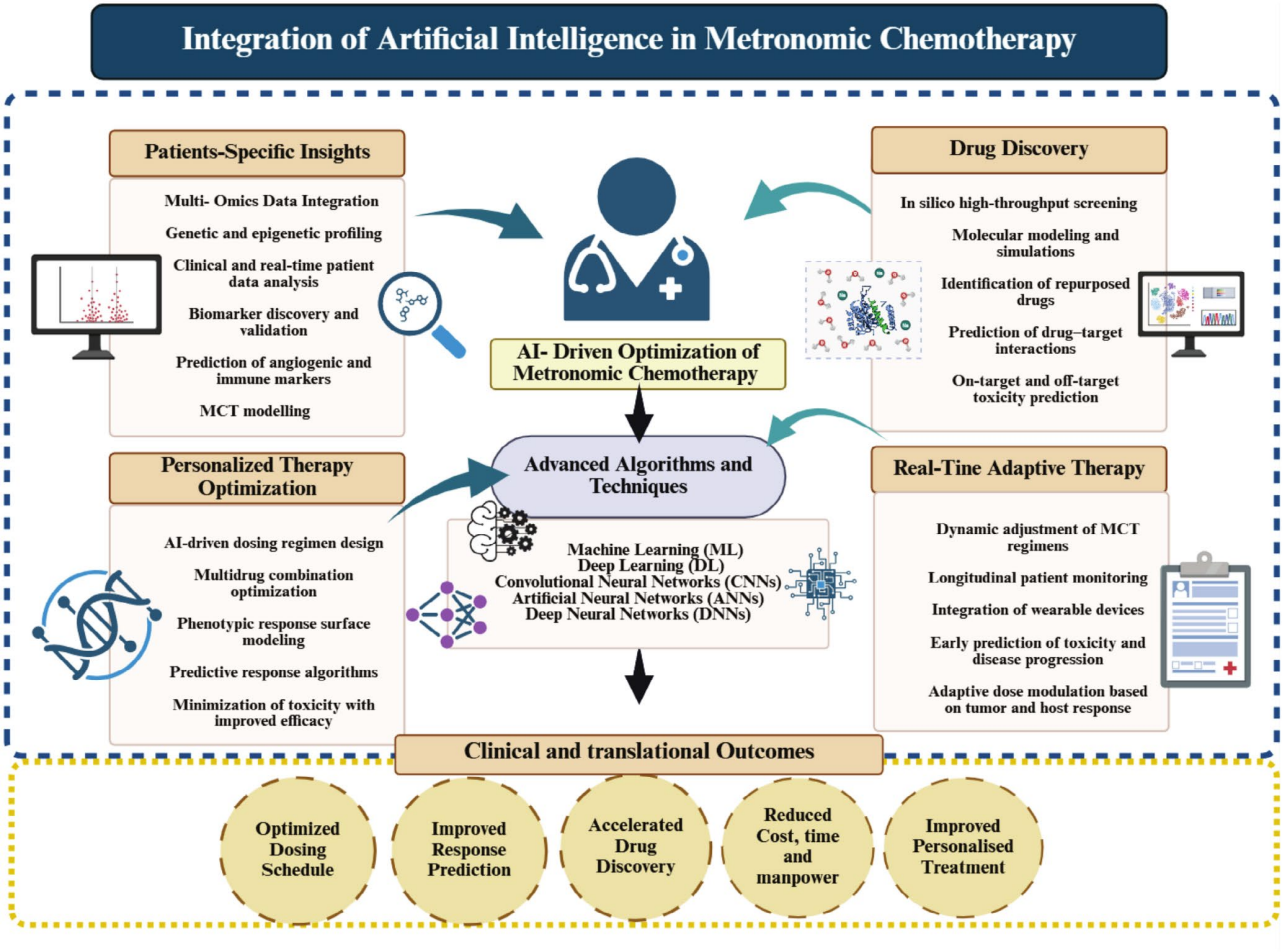
Integration of artificial intelligence (AI) in metronomic chemotherapy (MCT). Schematic representation illustrating how AI‐driven approaches integrate patient‐specific multi‐omics and clinical data with advanced machine learning and deep learning algorithms to optimize MCT. AI enables personalized dosing, optimization of drug combinations, real‐time adaptive therapy, and in silico drug discovery, leading to improved response prediction, reduced toxicity, accelerated therapeutic development, and enhanced personalized cancer treatment.

From a drug development perspective, AI‐driven platforms (Table 2) integrate molecular simulations, AlphaFold, protein structure databases (PDB), and chemical libraries (ZINC, PubChem, ChEMBL, and DrugBank) to predict protein‐drug interactions, model 3D protein conformations, evaluate binding affinities, and forecast on‐target and off‐target toxicities [82]. Natural language processing (NLP) and AI‐based predictive tools like PISTON can anticipate pharmaceutical side effects and optimize dosing regimens [80]. Large‐scale statistical analyses performed by AI algorithms further improve the reliability, interpretability, and reproducibility of experimental and clinical findings, supporting faster and more accurate hypothesis testing [78]. The integration of AI across genomics, proteomics, radiomics, clinical monitoring, and pharmacological modeling helps overcome the limitations of conventional therapies, enhances treatment tolerability, and contributes to improved clinical outcomes as illustrated in Figure 3. Collectively, these advances underscore the potential of AI to refine and personalize MCT in OC by optimizing dosing strategies, predicting therapeutic response, minimizing toxicity, and enabling adaptive, patient‐specific treatment approaches.

**TABLE 2:** Consolidated AI‐driven tool categories relevant to drug design, screening, and optimization for metronomic chemotherapy (MCT) in ovarian cancer.

AI tool category	Representative tools	Core algorithms	Primary function	Functional impact in AI‐driven MCT for ovarian cancer	References
ADMET and toxicity prediction	AdmetSAR, DeepTox, DeepCarc, eToxPred, LimTox, PrOCTOR, and Toxtree	SVM, RF, DNN, CNN, and decision trees	Prediction of absorption, distribution, metabolism, excretion, carcinogenicity, and organ toxicity	Enables selection of low‐toxicity compounds, supports chronic low‐dose scheduling, and minimizes cumulative toxicity critical for long‐term MCT	[86, 87, 88]
Physicochemical and PK property estimation	ALGOPS, EPI Suite, and PPB2	NN, QSPR, and Bayesian models	Prediction of solubility, lipophilicity, permeability, and protein binding	Supports oral bioavailability, dose stability, and consistent exposure required for metronomic regimens	[88]
QSAR and activity prediction	ANN/DNN‐QSAR, QSAR‐Co‐X, chemSAR, and VISAR	ANN, DNN, RF, and GBM	Structure–activity relationship modeling and feature interpretation	Identifies potent agents at sub‐MTD doses and reveals mechanistic drivers compatible with MCT	[89, 90, 91]
Drug‐target interaction (DTI) prediction	DeepDTA, DeepAffinity, DeepPurpose, WideDTA, and SimBoost	CNN, RNN, DL, and GBM	Prediction of binding affinity and interaction strength	Enables precise target engagement at low doses, improving efficacy without dose escalation	[78, 83]
Molecular docking and binding scoring	AutoDock Vina, Delta Vina, and ODDT	RF scoring and empirical scoring	Ligand‐target docking and binding energy estimation	Supports rational drug selection and prioritization of compounds effective under metronomic exposure	[89]
De novo drug design and chemical generation	Chemical VAE, Junction Tree VAE, LatentGAN, REINVENT, ORGANIC, and ReLeaSE	VAE, GAN, RNN, and reinforcement learning	Generation of novel drug‐like molecules	Facilitates discovery of novel low‐toxicity compounds optimized for sustained dosing	[82, 83]
Drug repurposing and network medicine	DrugNet, DRIMC, DeepDR, GIPAE, and RepCOOL	ML, Bayesian matrix completion, and DL	Drug‐disease and drug‐target network integration	Accelerates identification of approved or low‐dose compatible drugs for MCT combinations in ovarian cancer	[92, 93]
Metabolism and CYP liability prediction	XenoSite, SMARTCyp, and FAME	Neural networks	Prediction of CYP‐mediated metabolic hotspots	Enables dose fine‐tuning and avoidance of metabolic liabilities during prolonged MCT	[88]
Polypharmacology and target profiling	iDrugTarget and PPB2	ML, Naïve Bayes, and NN	Multi‐target interaction prediction	Supports multi‐pathway modulation, aligning with the multi‐target nature of MCT	[80]
Literature mining and pharmacovigilance	PISTON	NLP and topic modeling	Side‐effect detection and safety signal extraction	Enhances long‐term safety monitoring and risk mitigation in chronic MCT administration	[80]
AI‐assisted drug design pipelines	AMPL, PADME, HitDexter, and LigMate	RF, CNN, XGBoost, and DL	End‐to‐end molecular modeling and screening	Streamlines candidate prioritization for MCT‐compatible drug development	[81, 83]

## 9 AI‐Assisted Strategies to Enhance MCT

AI encompasses a broad spectrum of computational tools and algorithms that are increasingly explored for enhancing MCT through optimization of drug discovery, dosing strategies, toxicity prediction, and treatment personalization. By integrating ML, DL, NLP, and quantitative structural–activity relationship (QSAR/QSPR) modeling, AI enables the analysis of large pharmacological and clinical datasets to support the development of effective low‐dose therapeutic regimens central to MCT [94].

Moreover, AI‐driven predictive platforms and pharmacovigilance tools, such as PISTON, DeepChem, and eToxPred, can evaluate adverse drug reactions, pharmacokinetics, bioavailability, and cumulative toxicity, thereby improving safety assessment and rational drug selection for prolonged metronomic treatment schedules as summarized in Table 2 [79, 81]. In addition, AI‐enabled structure‐based and ligand‐based virtual screening approaches facilitate rapid identification and prioritization of candidate compounds with favorable low‐dose activity and therapeutic profiles suitable for metronomic strategies [81, 95].

Recent AI‐driven drug discovery studies across multiple disease models and cancer systems have demonstrated the potential of AI‐assisted technologies to optimize the therapeutic selection and drug repurposing, which may be adaptive to MCT. AI‐based QSAR and DL models such as Cloud 3D‐QSAR, PyQSAR, VISAR, and MproI‐GEN have shown utility in compound identification, mechanistic analysis, and candidate optimization through integration of molecular, structural, and pharmacological data [75, 89, 90, 96, 97]. These approaches may support identification of low‐dose agents and synergistic drug combinations suitable for continuous metronomic regimens.

Similarly, AI‐enabled platforms, including DeepChem, DeepPurpose, DrugCell, and ANN/DNN‐based predictive models, facilitate the evaluation of drug response, molecular interactions, and treatment sensitivity, thereby supporting personalized and adaptive therapeutic approaches [78, 83, 96]. In the context of OC, such technologies may help optimize metronomic combinations involving cyclophosphamide, bevacizumab, PARP inhibitors, or immunotherapeutic agents by improving patient stratification and predicting therapeutic response.

AI‐based pharmacokinetic and toxicity prediction tools, such as ALGOPS, EPI Suite, DeepCarc, eToxPred, SMARTCyp, XenoSite, and FAME, further contribute to optimization of dosing schedules, oral bioavailability, metabolism, and cumulative toxicity, which are critical considerations for prolonged low‐dose MCT administration [19, 86, 87, 88, 98]. In addition, ML‐driven repurposing platforms, including PREDICT, MANTRA, BiRWDDA, RepCOOL, SNF‐CVAE, and iDrug, may facilitate the identification of novel therapeutic combinations and adaptive treatment strategies relevant to metronomic oncology approaches [91, 92].

Cancer‐specific AI applications have also demonstrated translational potential. Li et al. developed a DL‐based repurposing strategy integrating chemical structures and transcriptomics data for non‐small cell lung cancer, an approach that may be extended to OC and MCT‐based precision therapy [93]. Similarly, predictive oncology tools such as mPS and ExPecto have shown utility in survival prediction and tissue‐specific gene expression modeling, potentially supporting adaptive metronomic scheduling in genetically stratified patient populations [99].

Importantly, recent clinical translation of AI‐assisted drug discovery, including the development of Exscientia AI‐designed A2A receptor antagonists and orally bioavailable CDK8 inhibitors, further highlights the potential of AI‐guided approaches for future integration into metronomic combination strategies [100]. Collectively, these AI‐assisted technologies provide a promising framework for enhancing MCT through optimization of dosing strategies, toxicity prediction, drug repurposing, biomarker‐guided patient selection, and personalized therapeutic approaches, particularly in OC management, as summarized in Table 2.

## 10 Discussion

OC continues to represent a major clinical challenge due to its late‐stage diagnosis, molecular heterogeneity, and frequent development of treatment resistance [1, 2]. Despite advances in surgical techniques and platinum‐based chemotherapy, long‐term survival remains limited, underscoring the urgent need for innovative, durable, and patient‐friendly therapeutic strategies [6]. The biological diversity of OC, including differences in histology, genomic instability, angiogenic dependence, immune evasion, and apoptotic dysregulation, necessitates a multimodal and adaptable treatment strategy [9, 40].

Recent years have witnessed a paradigm shift toward precision oncology with the incorporation of targeted therapies such as PARP inhibitors, anti‐angiogenic agents (e.g., bevacizumab), and immune checkpoint inhibitors. These approaches exploit specific molecular vulnerabilities, including homologous recombination deficiency and VEGF‐driven angiogenesis, enabling improved patient stratification and clinical outcomes [6, 10].

However, despite these advances, their long‐term efficacy is often limited by acquired resistance, cumulative toxicity, and high treatment costs, highlighting the need for alternative or complementary therapeutic strategies. Within this context, MCT has emerged as a biologically rational and clinically relevant approach. By employing continuous or frequent low‐dose drug administration, MCT shifts the therapeutic focus from direct tumor cytotoxicity to modulation of the tumor microenvironment. Evidence from preclinical and clinical studies indicates that MCT exerts antitumor effects through sustained inhibition of angiogenesis, suppression of EPC mobilization, immune modulation (including depletion of regulatory T cells and myeloid‐derived suppressor cells), and induction of tumor dormancy [13, 16].

These multimodal mechanisms distinguish MCT from conventional MTD chemotherapy and support its role as a long‐term disease control strategy. The relevance of MCT is especially pronounced in LMICs, where healthcare infrastructure, financial constraints, and limited access to high‐cost targeted therapies pose substantial challenges. Oral metronomic regimens reduce the need for hospitalization, infusion facilities, and intensive supportive care, thereby lowering overall treatment costs and improving accessibility. These attributes position MCT as a pragmatic and scalable strategy for global OC care [24, 56].

Beyond monotherapy, MCT is increasingly being explored in combination with immunotherapy and targeted agents. By reshaping the tumor microenvironment, normalizing tumor vasculature, reducing hypoxia, and alleviating immune suppression, MCT can enhance the efficacy of immune checkpoint inhibitors and anti‐angiogenic therapies [101]. Early‐phase clinical trials combining metronomic cyclophosphamide with bevacizumab and pembrolizumab have yielded encouraging results, supporting the concept of rational, biology‐driven combination regimens [69].

Parallel to these therapeutic advances, AI has begun to redefine the landscape of cancer research, drug discovery, and clinical decision‐making [86]. AI has the ability to integrate and analyze vast, heterogeneous datasets spanning genomics, transcriptomics, proteomics, pharmacology, imaging, and clinical outcomes and eventually offers unprecedented opportunities to optimize MCT strategies in OC [85]. ML, DL, QSAR modeling, and network‐based approaches are increasingly employed to identify novel drug candidates, predict drug target interactions, assess toxicity and pharmacokinetics, and uncover synergistic drug combinations [95]. AI‐driven platforms have demonstrated significant potential in accelerating drug repurposing, toxicity prediction, and de novo drug design, thereby improving the efficiency of the preclinical pipeline and reducing reliance on traditional experimental approaches [85, 94].

Furthermore, AI‐supported clinical trial designs may facilitate adaptive dosing strategies, improve patient selection, and enhance the likelihood of clinical success. These capabilities are especially relevant for MCT, where treatment efficacy depends on dynamic interactions between tumor biology, host response, and dosing patterns. Despite these promising advances, several limitations and challenges must be acknowledged. For MCT, prolonged drug exposures raise concerns regarding cumulative toxicity, including hematological suppression, fatigue, and potential long‐term organ effects. In addition, variability in dosing regimens, lack of standardized protocols, and limited large‐scale randomized trials hinder the establishment of MCT as a universally accepted standard of care. The identification of predictive biomarkers to guide patient selection also remains an unmet need.

Similarly, the integration of AI into oncology and MCT faces important challenges. These include issues related to data quality and standardization, limited availability of high‐quality annotated datasets, lack of algorithm transparency (“black box” models), reproducibility concerns, and regulatory and ethical considerations surrounding data privacy and clinical implementation [84]. Furthermore, the clinical translation of AI‐driven insights remains relatively slow, partly due to the need for rigorous validation and integration into existing healthcare systems.

Looking forward, future research should focus on generating high‐quality clinical evidence through well‐designed randomized trials evaluating MCT alone and in combination with targeted and immunotherapeutic agents. The development of standardized dosing protocols, identification of robust predictive biomarkers, and incorporation of patient‐reported outcomes will be critical to optimizing clinical utility. Concurrently, advances in AI should aim to improve model interpretability, integrate multi‐omics and real‐world data, and enable real‐time adaptive treatment strategies.

The convergence of MCT and AI represents a promising frontier in OC management. By combining biologically informed therapeutic strategies with data‐driven optimization, this integrated approach has the potential to enhance treatment precision, improve tolerability, and reduce healthcare costs. Ultimately, AI‐guided MCT may facilitate the transition of OC toward a more manageable chronic disease model, particularly in resource‐constrained settings where accessible and sustainable treatment options are urgently needed.

## 11 Conclusion

In summary, OC continues to present significant clinical challenges despite advances in current management strategies. Emerging developments in targeted therapy, immunomodulation, and MCT have shown encouraging potential and may contribute to the evolving therapeutic landscape of OC. MCT offers a biologically rational, cost‐effective, and patient‐centric approach that aligns well with the principles of precision oncology. Furthermore, the integration of AI into this framework amplifies its potential by accelerating drug discovery, enhancing treatment personalization, and improving clinical outcomes, as shown comprehensively in Figure 3. However, the clinical implementation of AI‐driven strategies in OC remains limited at present and requires further validation through robust preclinical and clinical studies. Continued research, interdisciplinary collaboration, and strategic investment in translational and AI‐enabled platforms will be important to better define the role of these emerging approaches and potentially improve survival outcomes, reduce treatment‐related toxicity, and enhance equitable OC care worldwide.

## Author Contributions

**Ravi Chauhan:** writing – original draft. **Ajaz A. Bhat:** writing – review and editing, conceptualization. **Rajeev Goyal:** writing – review and editing. **Shweta Tripathi:** writing – review and editing. **Ammira S. Al‐Shabeeb Akil:** writing – review and editing. **Lakshay Malhotra:** writing – original draft. **Shahab Uddin:** writing – review and editing. **Suraja Kumar Das:** writing – original draft. **Sameer Mirza:** writing – review and editing. **Khushi Nehra:** writing – review and editing. **Maysaloun Merhi:** writing – review and editing. **Mayank Singh:** conceptualization, writing – review and editing.

## Funding

This work was supported by funding from the Department of Biotechnology (DBT), Government of India, to Mayank Singh Extramural grant (Grant No. BT/PR40649/MED/31/441/2020). Sidra Medicine Precision Program provides research funding to Ajaz A. Bhat (SDR400190) and Ammira S. Al‐Shabeeb Akil (SDR400149).

## Ethics Statement

The authors have nothing to report.

## Consent

The authors have nothing to report.

## Conflicts of Interest

The authors declare no conflicts of interest.

## Data Availability

Data sharing is not applicable to this article as no datasets were generated or analyzed during the current study.
